# Intervention of electroacupuncture on spinal p38 MAPK/ATF-2/VR-1 pathway in treating inflammatory pain induced by CFA in rats

**DOI:** 10.1186/1744-8069-9-13

**Published:** 2013-03-22

**Authors:** Jian-Qiao Fang, Jun-Ying Du, Yi Liang, Jun-Fan Fang

**Affiliations:** 1 Department of Neurobiology and Acupuncture Research, The Third Clinical Medical College, Zhejiang Chinese Medical University, 548 Binwen Road, Binjiang District, Hangzhou, Zhejiang Province 310053, China

**Keywords:** Chronic inflammatory pain, Electroacupuncture, Anti-inflammatory pain, CFA, p38 MAPK, ATF-2, VR-1, COX-2, Signal transduction pathway

## Abstract

**Background:**

Previous studies have demonstrated that p38 MAPK signal transduction pathway plays an important role in the development and maintenance of inflammatory pain. Electroacupuncture (EA) can suppress the inflammatory pain. However, the relationship between EA effect and p38 MAPK signal transduction pathway in inflammatory pain remains poorly understood. It is our hypothesis that p38 MAPK/ATF-2/VR-1 and/or p38 MAPK/ATF-2/COX-2 signal transduction pathway should be activated by inflammatory pain in CFA-injected model. Meanwhile, EA may inhibit the activation of p38 MAPK signal transduction pathway. The present study aims to investigate that anti-inflammatory and analgesic effect of EA and its intervention on the p38 MAPK signal transduction pathway in a rat model of inflammatory pain.

**Results:**

EA had a pronounced anti-inflammatory and analgesic effect on CFA-induced chronic inflammatory pain in rats. EA could quickly raise CFA-rat’s paw withdrawal thresholds (PWTs) and maintain good and long analgesic effect, while it subdued the ankle swelling of CFA rats only at postinjection day 14. EA could down-regulate the protein expressions of p-p38 MAPK and p-ATF-2, reduced the numbers of p-p38 MAPK-IR cells and p-ATF-2-IR cells in spinal dorsal horn in CFA rats, inhibited the expressions of both protein and mRNA of VR-1, but had no effect on the COX-2 mRNA expression.

**Conclusions:**

The present study indicates that inhibiting the activation of spinal p38 MAPK/ATF-2/VR-1 pathway may be one of the main mechanisms via central signal transduction pathway in the process of anti-inflammatory pain by EA in CFA rats.

## Background

Inflammatory pain is a very common symptom in clinical practice. Patients mainly suffer from ongoing pain (spontaneous pain), evoked pain, and hyperalgesia. Among several types of chronic pains, chronic inflammatory pain has been recognized to be most common and difficult to treat. Therapy for inflammatory pain has been composed of symptomatic treatment with nonsteroidal anti-inflammatory drugs (NSAIDs), but long-term uses of this agent has shown side effects such as gastrointestinal toxicity and cardiovascular toxicity [1,2]. Thus, there is a need for anti-inflammatory pain treatment with less side effects.

P38 mitogen-activated protein kinase (MAPK) signal transduction pathway is typically activated by cellular stress and pro-inflammatory cytokines and plays a critical role in inflammatory responses. Systematic or intrathecal administration of p38 MAPK inhibitor has been shown to effectively alleviate inflammation and arthritis [3,4]. The activated p38 MAPK is translocated to the nucleus, where it can phosphorylate transcriptional factors such as ATF-2 [5]. The synthesis of several pro-inflammatory mediators such as cyclooxygenase-2 (COX-2), interleukin-1β (IL-1β), vanilloid receptor-1 (VR-1) in the spinal cord are up-regulated by p38 MAPK [6-8].

Electroacupuncture (EA), a Chinese medical therapy, is a modified acupuncture method which utilizes electrical stimulation and has been used to reveal the analgesic effects on acute pain and chronic inflammatory pain [9-11]. Clinical trials show that EA has beneficial effects in patients with various inflammatory diseases [12-14]. Experimental studies demonstrate that EA significantly inhibits complete Freund’s adjuvant- (CFA) or collagen-induced hind paw inflammation and hyperalgesia in a rat model [15-17]. In relation with EA analgesia and its mechanism in the inflammatory pain, there were lots of studies focusing on the analgesic effect of EA via opioid system and inflammatory mediators in CFA-induced and carrageenan-induced inflammatory pain models [18-21]. However, the underlying mechanisms of EA for inflammatory pain are still not completely understood. We previously reported that EA could attenuate CFA-induced inflammatory pain, at least in part, through suppressing numbers of p-p38 MAPK IR-cells in spinal cord [22]. In this study, we first used a rat CFA model to observe whether p38 MAPK signal transduction pathway in spinal cord plays an important role in inflammatory pain, and then to investigate whether EA may relieve inflammatory pain by counteracting the activation of p38 MAPK signal transduction pathway.

## Results

### Anti-inflammatory activity of EA

**Figure 1:**
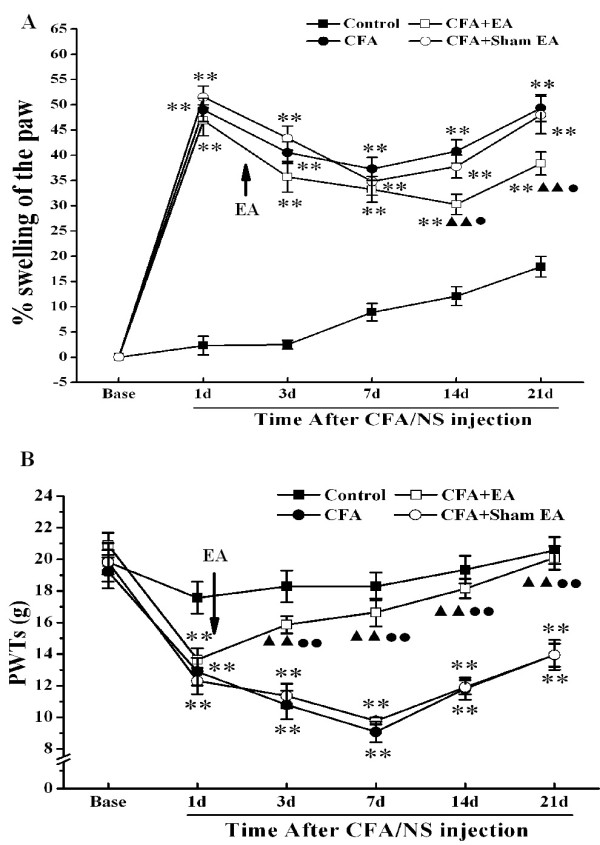
**Behavioral test of rats in each group at different time-point. A**. Ipsilateral percent swelling of the paw of rats in each group at different time-point. The percent swelling of the paw was assessed by using plethymometer and water displacement method. The percent swelling of the paw was measured pre-injection and on day 1, 3, 7, 14 and 21 after saline/CFA injection. All data are expressed as means±SEM, n=12. **P<0.01 vs. group Control. ^▲ ▲ ^*P*<0.01 vs. group CFA. ^● ^*P*<0.05, ^● ● ^*P*<0.01 vs. group CFA+Sham EA. **B**. Ipsilateral paw withdrawal thresholds (PWTs) of rats in each group at different time-point. PWTs were assessed by using dynamic plantar aesthesiometer. PWTs were measured pre-injection and on day 1, 3, 7, 14 and 21 after saline/CFA injection. All data are expressed as means±SEM, n=12. **P<0.01 vs. group Control. ^▲ ▲ ^*P*<0.01 vs. group CFA. ^● ● ^*P*<0.01 vs. group CFA+Sham EA.

As shown in Figure 1A, the percentage swelling of the paw in each group was evaluated by the water displacement plethysmometer at six time points, 1 day before and 1, 3, 7, 14, 21 days after saline/CFA injection. The repeated-measures ANOVA with between-subjects factors revealed differences in the percentage swelling of rat’s paw over days (*P*<0.01) and between groups (*P*<0.01). There was significant interactive effect between days and groups (*P*<0.01). Post-hoc LSD tests showed a significant difference between control group and other three groups (*P*<0.01). However, no difference in the paw edema was found between CFA group and CFA+Sham EA group. An anti-swelling effect was observed in the CFA+EA group when compared with that in CFA group (*P*<0.05) and CFA+Sham EA group (*P*<0.01). However, a significant difference in the paw edema was also found between control group and CFA+EA group (*P*<0.05).

For comparisons among groups at all time points, the results by One-way ANOVA for independent sample identified that the percentage swelling of the paw in three CFA injected groups were all markedly higher than that in control group at corresponding time points (*P*<0.01). However, CFA+EA group showed a significant lower score compared with the CFA and CFA+Sham EA groups at day 14 and day 21 after CFA injection.

### Analgesic activity of EA

Mean paw withdrawal thresholds (PWTs) for all experimental groups were shown in Figure 1B. The repeated-measures ANOVA with between-subjects indicated differences over days (*P*<0.01) and between groups (*P*<0.01). There was a significant interactive effect between days and groups (*P*<0.01). Post-hoc LSD tests indicated no significant difference in PWTs between CFA+Sham EA group and CFA group. However, the CFA+EA group showed an available hypoalgesic effect when compared with CFA group and CFA+Sham EA group (*P*<0.01). A significant difference PWTs was also found between control group and CFA+EA group (*P*<0.05).

To compare four groups at all time points, as shown in Figure 1B, no difference in basal PWTs among groups was observed before saline or CFA injection. PWTs in CFA group and CFA+Sham EA group were lower obviously than those in control group at corresponding time points (*P*<0.01), and reached its lowest at post-injection day 7. After EA stimulation, the rats’ PWTs were increased rapidly and significantly higher than those in CFA group and CFA+Sham EA group at day 3, 7, 14, 21 after CFA injection (*P*<0.01), and showed no difference with those in control group (*P*>0.05).

### CFA-induced p38 MAPK activation in the spinal cord

**Figure 2:**
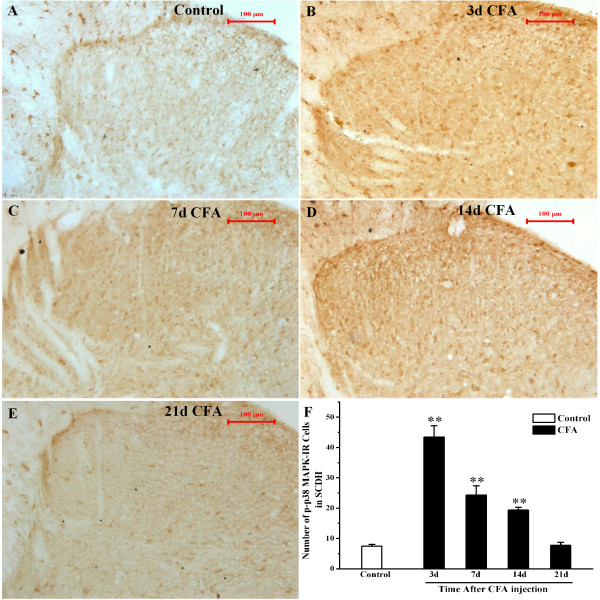
**CFA induces p38 MAPK activation in the ipsilateral spinal cord dorsal horn at different time-point. A-E**. Immunohistochemistry shows p-p38 MAPK IR cells in the L4-6 ipsilateral spinal cord dorsal horn of Control rats (**A**), CFA rats at 3 d (**B**), 7 d (**C**), 14 d (**D**) and 21 d (**E**). **F**. Quantification of p-p38MAPK IR shows that CFA induces p-p38 MAPK IR cells expression in the L4-6 ipsilateral spinal cord dorsal horn after day 3–14. **P<0.01 vs. group Control, n=3-5. All data are expressed as means±SEM. Scale bars: 100 μm, section thickness: 30 μm.

To investigate the possible over-expression of p-p38 MAPK activated by peripheral inflammation, we injected rat subcutaneously 0.1 ml CFA into the plantar surface of right hind paw. It induced a localized swelling and hypersensitivity to mechanical stimuli (Figure 1A-B), which persisted for 21 days through the duration of the experiment. An anti-p-p38 MAPK antibody was used to study the changes in p38 MAPK activation. P-p38 MAPK immunohistochemistry showed a low basal constitutive expression in the L4-6 spinal dorsal horn in rats of saline-injected control group (Figure 2A). The inflammation induced by CFA injection resulted in the induction of p-p38 MAPK in the dorsal horn on the ipsilateral side of L4-6 spinal cord (Figure 2). The increase was detectable and reached a peak at day 3 after CFA injection, and remained elevated with a slow decline until day 14. The most prominent increase of p-p38 MAPK IR cells was found in the laminas I-IV of the dorsal horn.

### CFA-induced ATF-2 activation in the spinal cord

**Figure 3:**
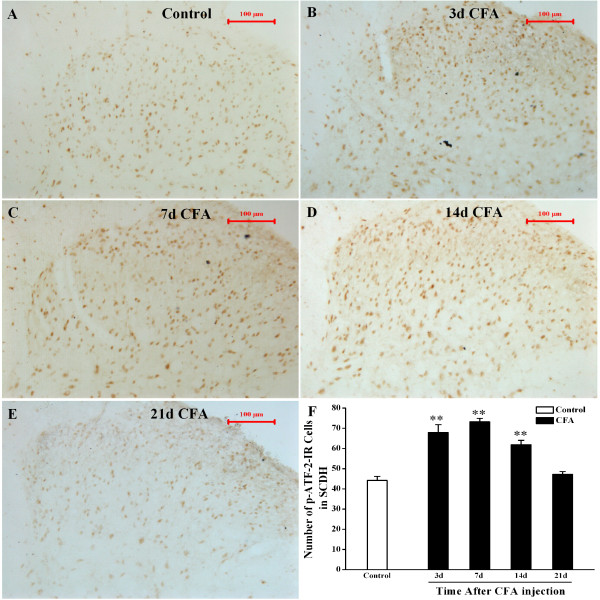
**Phosphor-ATF-2 IR cells expression in the Ipsilateral spinal cord dorsal horn in CFA rats at different time-point.** A-E. Immunohistochemistry shows p-ATF-2 IR cells in the L4-6 ipsilateral spinal cord dorsal horn of Control rats (**A**), CFA rats at 3 d (**B**), 7 d (**C**), 14 d (**D**) and 21 d (**E**) after CFA injection. **F**. Quantification of p-ATF-2 IR shows that CFA induces p-ATF-2 IR cells expression in the L4-6 ipsilateral spinal cord dorsal horn after day 3–14. **P<0.01 vs. group Control, n=3-5, All data are expressed as means±SEM. Scale bars: 100 μm, section thickness: 30 μm.

The changes of the activation of ATF-2 in spinal dorsal horn, in response to inflammation, were studied by immunohistochemistry. p-ATF-2 IR cells were also found in the laminas I-IV of the L4-6 spinal dorsal horn. A moderate basal constitutive expression of p-ATF-2 IR cells was observed in L4-6 spinal dorsal horn. CFA-injection induced a substantial increase in the number of p-ATF-2 IR cells in the ipsilateral dorsal horn (Figure 3). The increase was detected at day 3 after injection, reached a peak on day 7, and was maintained till day 14. On day 21, expression of p-ATF-2-IR cells almost returned to its basal constitutive level.

### Effect of EA on phosphor-p38 MAPK

**Figure 4:**
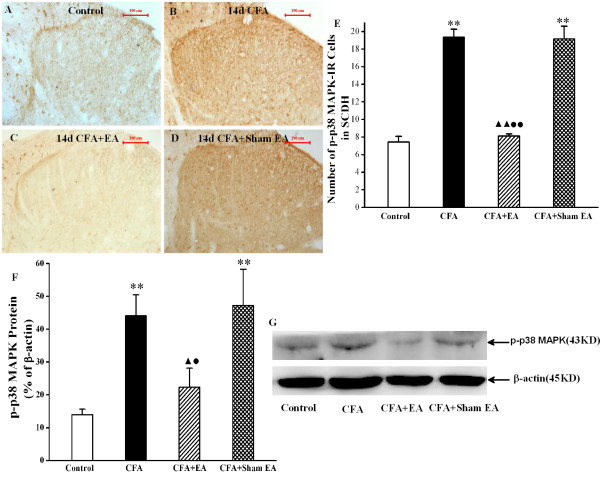
**Effect of EA on p-p38MAPK expression in the ipsilateral spinal dorsal horn at 14 day after CFA injection. A-E**. Immunohistochemistry shows p-p38 MAPK IR cells in the L4-6 ipsilateral spinal cord dorsal horn of Control rats (**A**), CFA rats (**B**), CFA+EA rats (**C**) and CFA+Sham EA rats (**D**) at 14 day after CFA injection. **E**. Quantification of p-p38 MAPK IR shows that EA suppresses p-p38 MAPK IR cells in the L4-6 ipsilateral spinal dorsal horn at 14 day after CFA injection. **P<0.01 vs. group Control. ^▲ ▲ ^*P*<0.01 vs. group CFA. ^● ● ^*P*<0.01 vs. group CFA+Sham EA. n=3-5. Scale bars: 100 μm, section thickness: 30 μm. **F**, **G**. Western blotting analysis reveals that EA suppresses p-p38 MAPK protein in the L4-6 ipsilateral spinal dorsal horn at 14 day after CFA injection. **G**. Quantification of F. p-p38 MAPK protein in normalized against β-actin. n=4-6, **P<0.01 vs. group Control. ^▲ ^*P*<0.05 vs. group CFA. ^● ^*P*<0.05 vs. group CFA+Sham EA.

As shown in Figure 4A-E, the numbers of p-p38 MAPK IR cells were increased in the L4-6 ipsilateral spinal dorsal horn on day 14 after CFA-injection, compared with that in saline group. After Sham EA treatment, the numbers of p-p38 MAPK IR cells were not decreased as compared with CFA group (*P*>0.05). While, stimulation of EA markedly decreased the numbers of p-p38 MAPK-IR cells, compared with that both in CFA group (*P*<0.01) and CFA+Sham EA group (*P*<0.01). We also used Western blotting method to detect p-p38 MAPK protein expression in the L4-6 ipsilateral spinal dorsal horn at day 14 after CFA-injection or saline-injection. It was observed that expressions of p-p38 MAPK protein in rats of CFA group and CFA+Sham EA group were much higher than that in rats of control group. EA significantly suppressed the expressions of p-p38 MAPK protein, compared with that in CFA group (*P*<0.05) and CFA+Sham EA group (*P*<0.05). In addition, the increased numbers of p-p38 MAPK IR cells in the superficial spinal dorsal horn was suppressed by the EA treatment at 3 d, 7 d and 14 d after CFA injection (P<0.01) (Additional file 1: Figure S1).

### Effect of EA on phosphor-ATF-2

**Figure 5:**
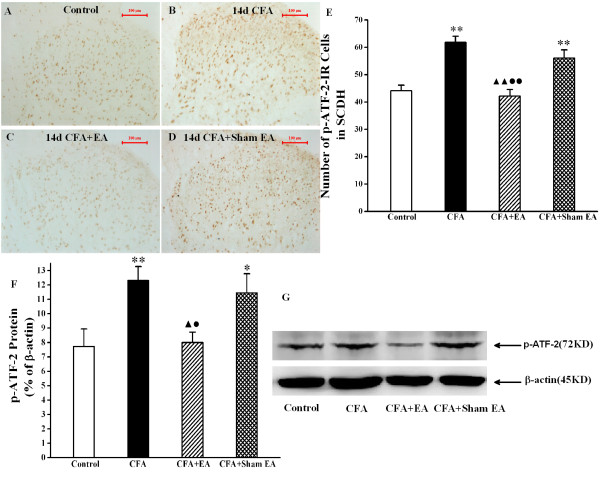
**Effect of EA on p-ATF-2 expression in the ipsilateral spinal dorsal horn at 14 day after CFA injection. A-E**. Immunohistochemistry shows p-ATF-2 IR cells in the L4-6 ipsilateral spinal cord dorsal horn of Control rats (**A**), CFA rats (**B**), CFA+EA rats (**C**) and CFA+Sham EA rats (**D**) at 14 day after CFA injection. **F**. Quantification of p-ATF-2 IR shows that EA suppresses p-ATF-2 IR cells in the L4-6 ipsilateral spinal dorsal horn on 14 day after CFA injection. **P<0.01 vs. group Control. ^▲ ▲ ^*P*<0.01 vs. group CFA. ^● ● ^*P*<0.01 vs. group CFA+Sham EA. n=3-5. Scale bars: 100 μm, section thickness: 30 μm. **F**, **G**. Western blotting analysis reveals that EA suppresses p-ATF-2 protein in the L4-6 ipsilateral spinal dorsal horn at 14 day after CFA injection. **F**. Quantification of **G**. p-ATF-2 protein in normalized against β-actin. n=4-6, *P<0.05, **P<0.01 vs. group Control. ^▲ ^*P*<0.05 vs. group CFA. ^● ^*P*<0.05 vs. group CFA+Sham EA.

The numbers of p-ATF-2 IR cells in the L4-6 ipsilateral spinal dorsal horn in rats of CFA group were greatly increased compared with that in control group on day 14 after CFA injection. After Sham EA treatment, the numbers of p-ATF-2 IR cells were not obviously decreased (*P*>0.05). However, stimulation of EA was able to markedly decrease the numbers of p-ATF-2 IR cells, compared either with CFA group (*P*<0.01) or with CFA+Sham EA group (*P*<0.01) (Figure 5A-E). Meanwhile, we applied western blotting method to detect p-ATF-2 protein expression in the L4-6 ipsilateral spinal dorsal horn in rats at day 14 after CFA-injection. It was shown that expressions of p-ATF-2 protein in rats of CFA group and CFA+Sham EA group were much higher than that in rats of saline group. EA strongly inhibited the expression of p-ATF-2 protein, compared with that in CFA group (*P*<0.05) and CFA+Sham EA group (*P*<0.05) (Figure 5F-G). In addition, we also tested the effect of EA on ATF-2 activation at 3 d, 7 d, 14 d and 21 d after CFA injection. As the same as p38 MAPK, the increased numbers of p-ATF-2-IR cells in the superficial spinal dorsal horn were suppressed by the EA treatment at 3 d, 7 d and 14 d after CFA injection (P<0.01) (Additional file 1: Figure S2).

### Effect of EA on COX-2 mRNA and protein expressions

**Figure 6:**
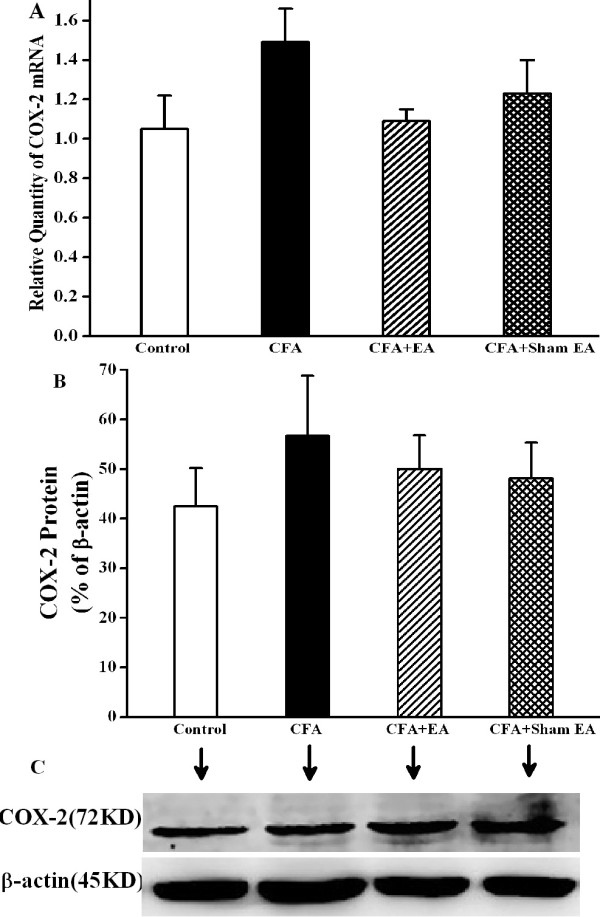
**Effect of EA on COX-2 expression in the ipsilateral spinal dorsal horn at 14 day after CFA injection. A**. Expression of COX-2 mRNA (relative to GAPDH) in L4-6 ipsilateral spinal cord dorsal horn measured by qPCR. Standard curve of COX-2 and GAPDH mRNA in the ipsilateral spinal cord dorsal horn is shown in Additional file 1: Figure S3. It shows that relative quantity of COX-2 mRNA has no significant difference among Control group, CFA group, CFA+EA group and CFA+Sham EA group on day 14 after CFA injection. n=5-7. **B**, **C**. Western blotting analysis reveals that COX-2 protein in the L4-6 ipsilateral spinal dorsal horn has also no significant difference among Control group, CFA group, CFA+EA group and CFA+Sham EA group on 14 day after CFA injection. **C**. Quantification of **B**. COX-2 protein in normalized against β-actin. n=4-6.

In the control group, COX-2 mRNA was expressed in the L4-6 ipsilateral spinal dorsal horn at low level. At day 14 after CFA injection, relative quantity of COX-2 mRNA expression of CFA group was increased, but had no significant difference with control group. After EA stimulation, relative quantity of COX-2 mRNA expression seemed less than that of CFA group and CFA+Sham EA group, but without significant difference (Figure 6A). We further detected COX-2 protein in the L4-6 ipsilateral spinal dorsal horn at 14th day after CFA injection. It was found that expressions of COX-2 protein showed no big difference among the three experimental groups (Figure 6B-C).

### Effect of EA on VR-1 mRNA and protein expressions

**Figure 7:**
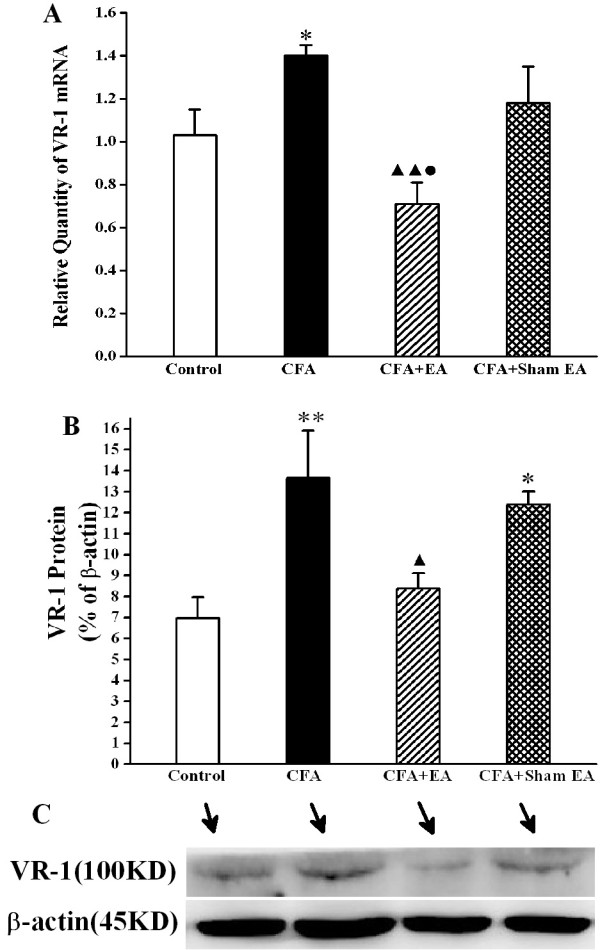
**Effect of EA on VR-1 expression in the ipsilateral spinal dorsal horn at 14 day after CFA injection. A**. Expression of VR-1 mRNA (relative to GAPDH) in L4-6 ipsilateral spinal dorsal horn measured by qPCR. Standard curve of VR-1 and GAPDH mRNA in the ipsilateral spinal dorsal horn is shown in Additional file 1: Figure S4. It shows that relative quantity of VR-1 mRNA of CFA group is much more than that of Control group on day 14 after CFA injection, and EA suppresses the expression of VR-1 mRNA. *P<0.05 vs. group Control. ^▲ ▲ ^*P*<0.01 vs. group CFA. ^● ^*P*<0.05 vs. group CFA+Sham EA. n=5-7. **B**, **C**. Western blotting analysis reveals that VR-1 protein in the L4-6 ipsilateral spinal cord dorsal horn has significant difference between Control group and CFA group, and EA suppresses the expression of VR-1 protein. **C**. Quantification of B. VR-1 protein in normalized against β-actin. *P<0.05, **P<0.01vs. group Control. ^▲ ^*P*<0.05 vs. group CFA. n=4-6.

On day 14 after CFA injection, relative quantity of VR-1 mRNA expression in the L4-6 ipsilateral spinal dorsal horn in rats of CFA group was significant higher than that of control group. Sham EA treatment reduced the expression of VR-1 mRNA to some extent, but showing no marked decrease as compared with CFA group. EA stimulation could remarkably down-regulate expression of VR-1 mRNA, compared with either CFA group or CFA+Sham EA group (Figure 7A). VR-1 protein expression in the L4-6 ipsilateral spinal dorsal horn was also delected at 14 day after CFA injection or saline injection. VR-1 protein was expressed in the control group at low level. However, there was highly expressed VR-1 protein in the CFA group and CFA+Sham EA group. The over-expressed VR-1 protein was obviously down-regulated by EA stimulation.

## Discussion

As a complementary and alternative medicine, EA has been accepted worldwidely, mainly for the treatment of acute and chronic pains. It is well know that EA has a good effect of anti-inflammation and analgesia. From this study, we found that the analgesic effect of EA had the property of a rapid onset, long duration and being strengthened along with EA treatment times, which was consistent with our previous report [22]. Interestingly, it was also found that the role of anti-inflammatory and analgesic effect of EA was not synchronistic. After repeated EA administration, quite a strong difference in the PWTs were found on day 3 between CFA+EA group and CFA group, and statistically significant difference was observed from day 3 to day 21. However, the onset of anti-inflammatory effect of EA appeared later than its analgesic effect. It was not until 14 treatments with EA, we firstly observed the difference in the paw edema of rats in CFA+EA group and that of CFA group. It could be predicted that EA got quicker analgesic effect, maybe through mediating some other way, not or not only by intervening on pro-inflammatory mediators in the treatment of inflammatory pain. Although EA analgesia is well documented, its mechanisms have not been thoroughly clarified. The present study demonstrates, for the first time, that EA suppressed the expression of p-p38 MAPK and its downstream p-ATF-2 as well as gene and protein expressions of VR-1 in the spinal dorsal horn, but had no inhibitory effect on gene and protein expressions of COX-2. Therefore, inhibition of the p38 MAPK activation may be the one of central pain sensitization mechanisms of EA analgesia for inflammatory pain.

The MAPK family includes p38 MAPK, extracellular signal regulated kinase (ERK1/2), c-jun N-terminal kinase (JNK), ERK7/8, ERK3/4 and ERK5 [23]. Initiation of the p38 MAPK cascade involves activation through a classic MAPK kinase kinase (MAP3K) – MAP kinase kinase (MKK) pathway. The p38 MAPK pathway is activated by a wide range of environmental or cellular stresses and proinflammatory cytokines and plays important roles in cell death, neurodegeneration, and inflammation [3]. Recently, p38 MAPK activation was shown to contribute to nociceptive responses in the spinal dorsal horn and dosal root ganglion (DRG) following inflammation and/or nerve injury. Following nerve injury, p-p38 MAPK levels sequentially increased in neurons, microglia, and astrocytes of the spinal dorsal horn; In addition, nerve injury-induced p-p38 MAPK occurred early and was long lasting [24-27]. Moreover, intrathecal injection of p38 MAPK inhibitors attenuated nociceptive response in different neuropathic pain animal models [25,28-31]. Several reports also indicated that a very robust increase of p-p38 MAPK in spinal dorsal horn induced hindpaw inflammation [4,6,32-34]. For example, bee-venom induced rapid increase in p-p38 MAPK in the spinal: p-p38 MAPK began to increase at 1 hour, reached a peak on day 3, and then decreased to normal level on day 7 [32]. In particular, different laboratories have shown that p-p38 MAPK was increased in spinal cord by CFA [4], formalin [6], capsaicin [33], or carrageenan [34]. Besides, intrathecal administration of the p38 inhibitor prevented inflammation-induced thermal and mechanical hypersensitivity [4,32]. In present study, we demontrated that the numbers of p-p38 MAPK-IR cells in spinal dorsal horn increased and reached at peak at day 3 after CFA injection, maintained for 14 days. The level of p-p38 MAPK protein expression in CFA group have returned to the base at day 21 after CFA-injection, however, inflammation and pain were still existed at that moment. The reason for this is unclear and is worthy of further study.

It is indicated that p38 MAPK is involved in the control of VR-1 mediated glioma apoptotic cell death [8]. Another study reports that p38 MAPK activated in the DRG following peripheral inflammation, by increasing VR-1 levels in nociceptive peripheral terminals, contributes to the maintenance of inflammatory pain [35]. P38 MAPK can potentially regulate protein expression in several different ways, activating transcription factors such as eukaryotic translation initiation factor 4E (eIF4E), Ets likely kinase (ELK-1), and cAMP response element binding protein (CREB) to increase transcription [36-38], increasing mRNA stability [39] or increasing translation. One major target of p38 MAPK is the translation factor ATF-2. ATF-2 (originally called CRE-BP1) is a member of the ATF/CREB family of transcription factors and is characterized by a basic zipper domain that consists of basic amino acids and a leucine zipper region, which acts as a DNA-binding region. The ATF-2 subfamily contains three members: ATF-2, CRE-BPa, and ATF-7 (originally known as ATF-a) [40]. ATF-2 regulates gene expression via the binding of ATF-2, as a homodimer, to the recognition sequences of the cAMP-response element (CRE) or via formation of a heterodimer with activating protein-1 (AP-1) that binds to CRE sequences. Thus, ATF-2 can interact with other members of the ATF family and with members of the AP-1 family [41]. It has been shown that p-p38 MAPK is involved in regulation of ATF-2 [42], which then binds to DNA and turns on the target gene [43]. In current report, we found that the numbers of p-p38 MAPK-IR cells at the same time coincided with the numbers of p-ATF-2-IR cells in spinal dorsal horn. So we presumed that ATF-2 as the downstream of p38 MAPK may play an important role in inflammatory pain.

It is now realized that a certain members of the transient receptor potential (TRP) receptors are key molecular integrators of the initiation and maintenance of pain. Vanilloid receptor-1 (VR-1), known as the transient receptor potential ion channel TRPV1, is essential for generating and transmitting pain [44,45]. It has been found to be expressed within those components of the peripheral and central nervous systems involved in pain detection, transmission and regulation [46]. It is all known that VR-1 can be found in a large population of primary sensory neurons, within the spinal cord itself, and within the brain. It has been reported that deletion of VR-1 is important for mediating thermal hyperalgesia and inflammatory swelling after CFA-induced inflammation [47]. This study was supported by other complementary studies which showed that the severity of arthritis in rodent models was reduced when VR-1 was either blocked or deleted [48]. VR-1 also plays an important role in mechanical hyperalgesia of inflammatory pain. When wild-type and VR-1 null mice are injected in one knee joint with CFA, the VR-1 null mice develops mild joint swelling (evidencing edema) and reduced mechanical hypersensitivity as compared with wild-type animals [49]. In present study, periphery inflammation induced a high expression of VR-1 in ipsilateral spinal dorsal horn on 14 day. EA could down-regulate the expressions of both VR-1mRNA and protein in spinal dorsal horn with increased PWTs.

Several factors have verified that p-p38 MAPK could up-regulate many proinflammatory mediators such as COX-2, IL-1β and VR-1 [6-8]. It has been demonstrated that local induction of COX-2 at the site of peripheral and the subsequent release of prostaglandin E_2_ (PGE_2_) has major roles in peripheral pain sensitization, which alters the threshold and excitability of the nociceptive peripheral terminal [50]. In addition to this peripheral action, COX-2 and prostaglandin also have a central function. The central PGE_2_ produced by COX-2 induced an electrophysiological transmitter release, direct depolarization of postsynaptic membranes and inhibiting glycine receptor activation [50]. The upregulation of central COX-2 level, induced by peripheral inflammatory stimuli such as CFA [51] and carrageenan [52], was linked to inflammatory allodynia and hyperalgesia. However, COX-2 just plays an important role in the development, but not maintenance of inflammation. Spinal COX-2 mRNA increased to a peak at 4 h after a intraspinal injection with IL-1α [53] and similarly increased to a peak at 4 h [53] or 8 h [54] after mechanical injury to the spinal cord. The expression of COX-2 protein also increased in the spinal cord during hindpaw inflammation at 4–6 h induced by CFA, paralleled the COX-2mRNA, and returned to baseline within 3 d after induction of inflammation [55]. According to another report, the expression of COX-2 mRNA on the ipsilateral side of spinal cord obtained from CFA injection was significantly increased at both 6 hour and 3 day after CFA injection compared with that from the saline-treated mice [56]. In agreement with above studies, we detected that the level of COX-2 mRNA in ipsilateral spinal dorsal horn was increased respectively at 5 hour and 3 day (Additional file 1: Figure S5), and returned to baseline within 14 day after CFA-induction of inflammation. Whereas, EA treatment did not suppress the increase of COX-2 mRNA expression in spinal dorsal horn at 5 hour, 3 day and 14 day.

As we mentioned above, robust p38 MAPK activation was essential for VR-1 and COX-2 expression in SCDH after peripheral inflammation, and it could regulate ATF-2 phosphorylation in SCDH of CFA-induced inflammation. Our previous report has demonstrated that EA analgesia and anti-inflammation were associated with its inhibition of spinal p38 MAPK activation [22]. In agreement with our previous study, pre-eletroacupuncture treatment has prophylactic analgesic effects on rats suffering from visceral pain by suppressing the local and spinal p38 MAPK [57]. The p38 MAPK signal transduction pathway is partly involved in the regulatory mechanism of this analgesic effect. Another study has reported that EA could improve immune suppression involving in the signaling pathway of p38 MAPK [58]. In this study, we first systemically investigated whether EA treatment might regulate the downstream materials of p38 MAPK. We found that EA did suppress the activation of the p38 MAPK and its downstream p-ATF-2 as well as VR-1mRNA and protein, but had no influence on gene and protein expressions of COX-2 at day 14 after CFA injection. Similarly, this study showed significant analgesic effect of EA at day 14 after CFA-injection, as well as a relative weak anti-inflammatory effect. PWTs of CFA+EA group had no difference with that of control group on day 14 in CFA-induced inflammatory pain, but the percent paw swelling of CFA+EA group had remarkable difference with that of control group at the same time. It could be suggested that both COX-2 and VR-1 play essential roles in inflammatory pain, however, EA just regulates the expressions of VR-1 mRNA and protein, and does not have obvious effect on COX-2 mRNA and protein expressions. It is well known that COX-2 mainly regulates the release of inflammatory mediators, leading to the generation of inflammatory pain, and VR-1 enhances the excitability of nerve resulting in pain [59,60]. Therefore, we speculate that the analgesic effect of EA, different from the mechanism of NASIDs, is not reached by inhibiting the release of inflammatory cytokines but rather by regulating pain sensitization, because we have not observed a suppressing effect of EA on COX-2 mRNA and protein, but an inhibitory effect on p38 MAPK, ATF-2 and VR-1 expressions.

## Conclusions

It is the feature of EA treatment that the desynchronized effect of analgesia and anti-inflammation exists when applied to alleviate inflammatory pain. EA can significantly attenuate CFA-induced inflammatory pain, which, at least in part, is contributed to its regulation of p38 MAPK/ATF-2/VR-1 pathway but not of p38 MAPK/ATF-2/COX-2 pathway in the persistent phase of inflammatory pain. In addition, EA could be a good complementary and alternative medicinal therapy in clinic for treatment of chronic inflammatroy pain.

## Methods

### Animal

In total, 126 male Sprague–Dawley rats (Department of Animal Sciences China), weighing between 220–240 g, were used for this study. They were housed six per cage on a 12-h light/dark cycle with controlled temperature and free accessed to food and water. Efforts were made to minimize animal discomfort and reduced numbers of animals used. All rats were used strictly in accordance with the National Institutions of Health Guide for the Care and Use of Laboratory Animals.

### Model establishment and experimental groups

Inflammatory pain model was established by injecting subcutaneously with 0.1 ml Complete Freund’s Adjuvant (CFA, Sigma, USA) into right hindpaw of rats.

The rats were separated randomly into 4 groups: 1) control group, 0.1 ml saline injection as used for CFA injection; 2) CFA group, 0.1 ml CFA injection, immobilization; 3) CFA+EA group, 0.1 ml CFA injection, immobilization and with EA stimulation; 4) CFA+ Sham EA group, 0.1 ml CFA injection, immobilization and with Sham EA stimulation.

### EA treatment

The EA treatment procedure was the same as reported in our previous study [22]. Rats were loosely immobilized by assistants’ hands. Four stainless steel acupuncture needles of 0.25 mm in diameter were inserted a depth of 5 mm into bilateral “Zusanli” (ST36, 5 mm lateral to the anterior tubercule of the tibia) and “Kunlun” (BL60, at the ankle joint level and between the tip of the external malleolus and tendo calcaneus) acupoints. It has been showed that these two acupoints were good at treat inflammatory pain [61]. The two ipsilateral needles were connected with the output terminals of the HANS Acupuncture point Nerve Stimulator (LH-202H, Huawei Co., Ltd., Beijing, China). The EA parameters were set as follows: square wave current output (pulse width: 0.2 ms); intensities ranging from 1–2 mA (each intensity for 15 min, totaling 30 min); at a 100 Hz and 2 Hz alternating frequencies (automatically shifting between 100 Hz and 2 Hz stimulation for three seconds each) [62]. The stimulation was given for 30 min, once per day, and started on day 1 after CFA injection when the assessment of behavioral test has been finished. Sham EA group animals received needle insertion subcutaneously into ST36 and BL60, but without electrical stimulation.

### Behavioral testing

Twelve rats of each group were included for assessment of behavioral test, which consist of paw volume and paw withdrawal thresholds (PWTs). Paw volume and PWTs were measured before CFA/saline injection (as Base) and 1 day after CFA/saline injection (before EA stimulation), 3, 7, 14 and 21 day after CFA/saline injection (after EA stimulation).

### Paw volume

Paw volume was measured by a water displacement plethysmometer (7140, UGO Basile, Italy). The right hindpaw was immersed in a chamber containing electrolyte solution up to the boundary between hairy and nonhairy skin, and the volume displacement was determined electronically. The percentage swelling of the paw was calculated by the following formula: % swelling= (V_t_-V_0_)/V_0_×100, where V_t_ is the paw volume after CFA/saline injection and V_0_ is basal paw volume.

### PWTs

Rats were placed on a metal mesh table and adapted to the new environment. The mechanical stimulus was delivered to the plantar surface of right hind paw from below the floor of the test chamber by an automated testing device (dynamic plantar aesthesiometer 37450, UGO Basile, Italy). A steel rod (diameter of 0.5 mm) was pushed against the hind paw with ascending force. The force went from 0 to 50 g over a 20 s period. When the animal withdrew its hind paw, the mechanical stimulus was automatically stopped, and the force at which the animal withdrew its paw was recorded as PWTs. Withdrawal responses were taken from four consecutive trials with at least 3min between trials and averaged.

### Perfusion and tissue harvest

Animals were sacrificed after 3, 7, 14 and 21 days post-model respectively. Rats were administered 10% choral hydrate in a volume of 0.35 mL, i.p., per 100 g body weight. Five animals of each group were perfused for immunohistochemical analysis. Once animals were deeply anesthetized, they were quickly perfused with 150 ml cold sterilized saline followed by 500 ml cold, fresh 4% paraformaldehyde in 0.1 M phosphate-buffered saline (PBS). The L4-6 segments of spinal cord were removed and postfixed in the same fixative for 6 h at 4°C before transfer to 15%, 30% sucrose for cytoprotection. Tissue was then embedded in an optimum cutting temperature medium, cut using a cryostat at a thickness of 30 μm and processed as free floating sections. Thirteen animals of each group were perfused with ice cold saline to clear contaminating blood. The same region of spinal cord as that used for western blot and qPCR snap frozen in liquid nitrogen and stored at −80°C.

### Immunohistochemistry

All of the sections were blocked with 5% goat serum in TBST (0.1%Tween-20) for 1 h at 37°C and incubated over one nights at 4°C with primary antibodies (diluted in 5% BSA/TBST), The following primary antibodies were used: rabbit anti-rat p-p38MAPK (1:200, CST, USA), rabbit anti-rat p-ATF-2 (1:200, CST, USA). The sections were then incubated for 1 h at 37°C with HRP-conjugated-avidin (1:400). Following incubation with HRP-conjugated-avidin (1:400) for 1 h at 37°C, sections were incubated with DAB substrate for 30 s. The stained sections were examined with a Leica microscope, and images were captured with a CCD camera. The number of p-p38 MAPK and p-ATF-2 positive cells was counted automatically five regions in a given area of upper left, lower left, upper right, lower right and middle using Image Pro Plus 6.0 under blinded conditions and averaged. A minimum of five tissue sections/animal with a minimum of three-five animals/group were counted.

### Western blotting

Samples were kept at −80°C until manually homogenized with liquid nitrogen and added to RIPA buffer (1% sodium deoxycholate, 0.1% SDS, sodium orthovanadate, sodium fluoride, EDTA, leupeptin, 150 mM NaCl,1% Triton X-100,50 mM Tris, pH 7.4) containing protease inhibitors, phosphatase inhibitor. The homogenate was allowed to rest on ice for 30 min and was then centrifuged at 15,000 rcf for 15 min at 4°C, and the supernatant collected. The protein concentration of tissue lysates was determined with a BCA protein Assay Kit, and 30 μg of protein was loaded in each lane. Protein samples were separated on 5%-10% SDS-PAGE gel and electrophoretically transferred to nitrocellulose membranes (Millipore, USA). The membranes were blocked with 5% low-fat milk in TBST for 1 h at room temperature. We used rabbit anti-rat p-p38 MAPK (1:1000, CST, USA), rabbit anti-rat p-ATF-2 (1:1000, CST, USA), rabbit anti-rat COX-2 (1:300, Cayman, USA), rabbit anti-rat VR-1 (1:200, Santa Cruz, USA) as primary antibodies and horseradish peroxidase (HRP)-conjugated goat anti-rabbit IgG as secondary antibody (1:10000). Rabbit anti-rat β-actin (1:1000, CST, USA) was used as internal control. The membranes were developed with ECL kit (Pierce, USA) and the signals were captured with Image Quant LAS 4000 (GE, USA). Scanned images were analyzed by Image Quant TL7.0 Analysis Software (GE, USA).

### qPCR

After tissue homogenization in ice-cold tubes, total RNA was extracted using Trizol (Invitrogen, France). Reverse transcription was performed using 1 μg total RNA using the PrimeScript® RT reagent Kit With gDNA Eraser (TaKaRa, Japan). Expression of COX-2 and VR-1 genes was analyzed by real-time quantitative polymerase chain reactions (qPCR) using the CFX96™ real-time PCR detection system (Bio-Rad, USA). The glyceraldehyde-3- phosphate dehydrogenase (GAPDH) gene served as an internal control for expression levels of target genes. Primers were synthesized to amplify a gene segment of GAPDH of 288 base pairs (bp): 5^′^-TGCTGAGTATGTCGTGGAG-3^′^(sense), 5^′^-GTCTTCTGAGTGGCAGTGAT-3^′^(anti-sense). A 161-bp segment of the COX-2 gene was amplified with 5^′^-CACGGACTTGCTCACTTTGTT-3^′^ (sense), and 5^′^-AAGCGTTTGCGGTACTCATT-3^′^ (anti-sense). A 129-bp segment of the VR-1 gene was amplified with 5^′^-GAAAGGATGGAACAACGGGCGC-3^′^ (sense), and 5^′^-GAAGATGTGGGGCTTGACTGG-3^′^ (anti-sense). The primers were designed based on sequences from the Genebank database. EvaGreen (Bio-Rad, USA) served as a dye that binds to amplified DNA to emit fluorescence during reactions. EvaGreen recently emerged as an optimal green fluorescent DNA dye for qPCR, it has equal or better sensitivity compared with SYBR Green I. The reaction mixture of 20 μl contained 10 μl SsoFast EvaGreen supermix (Bio-Rad, USA), o.4 μl sense and anti-sense primers (400 nM), 1.5 μl template complementary DNA, 7.7 μl Rnase/Dnase-free water. Reactions (total volume, 20 μl) were incubated at 95°C for 30 m, followed by 40 cycles of 10 s at 95°C and 30 s at 55.9°C (COX-2 mRNA) / 59.0°C (VR-1 mRNA). Water controls were included to ensure specificity. Each sample was measured in triplicate, and data points were examined for integrity by analysis of the amplification plot. Adding the melting curve analysis in the reaction condition, the analytical model: 65°C-95°C, an increase of 0.5°C every 10 s. The comparative cycle threshold Cq method was used for relative quantification of gene expression. The amount of COX-2 mRNA and VR-1 mRNA, normalized to the GAPDH and relative to a calibrator, was given by 2^-ΔΔCq^, with Cq indicating the cycle number at which the fluorescence signal of the PCR product crosses an arbitrary threshold set with the exponential phase of the PCR.

### Statistical analysis

All data were expressed as means ± standard error mean (SEM). The PWTs and the percentage swelling of paw change data were normally distributed, and were therefore analyzed using repeated-measures ANOVA with between-subjects factors. A one-way ANOVA for independent samples compared differences between groups at each time. Post hoc testing was performed with a Fisher’s PLSD test for differences between groups. The criterion for statistical significance was *P*<0.05.

## Competing interests

The authors declare that they have no competing interests.

## Authors’ contributions

JQF designed and performed experimental protocols described in this manuscript as well as the writing of the initial draft of the manuscript. JYD performed the immunohistochemistry and Western blotting, tissue fractionation and associated analyses. YL provided supervision for data analysis, study direction, image acquisition, manuscript design and revisions. JFF performed experiments, contributed to the design, data analysis and writing of the manuscript. All of the authors have read and approved the final manuscript.

## Supplementary Material

Additional file 1 Effect of EA on p38 MAPK pathway at different time in CFA rats. Click here for file
