# Supplementary material for: Intervention of electroacupuncture on spinal p38 MAPK/ATF-2/VR-1 pathway in treating inflammatory pain induced by CFA in rats
Source: Mol Pain. 2013 Mar 22;9:13. doi: 10.1186/1744-8069-9-13 (PMC3608238; doi:10.1186/1744-8069-9-13)
Supplement: Additional file 1 — Effect of EA on p38 MAPK pathway at different time in CFA rats. [file 1744-8069-9-13-S1.doc]

**Effect of EA on p38 MAPK pathway at different time in CFA rats**

**Supplementary figures**


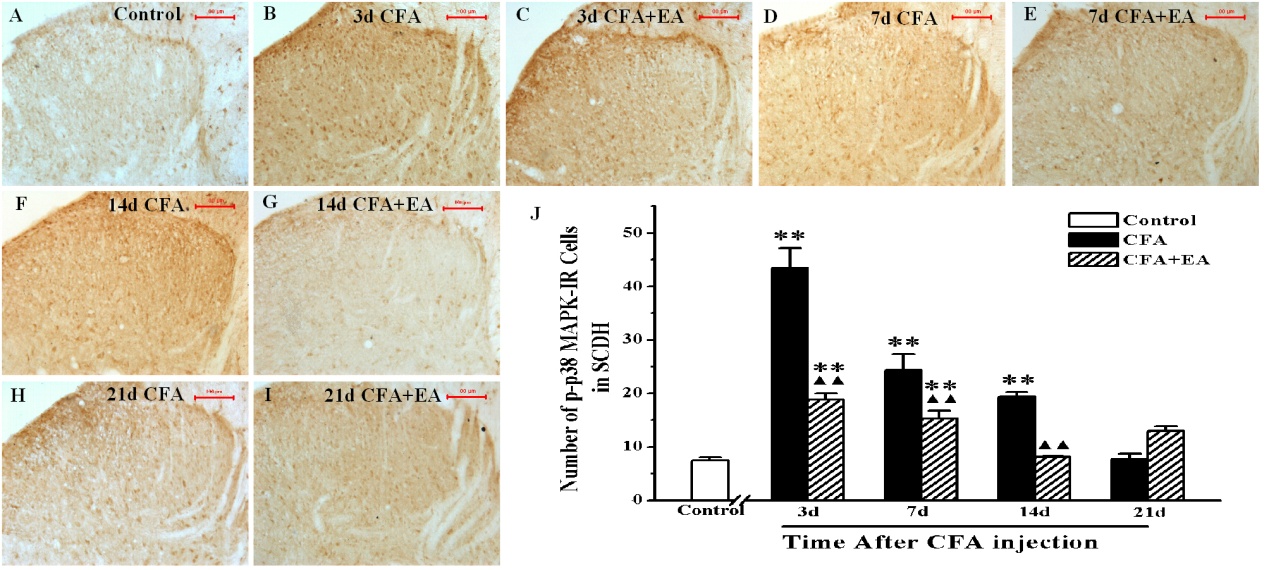


Supplementary figure 1


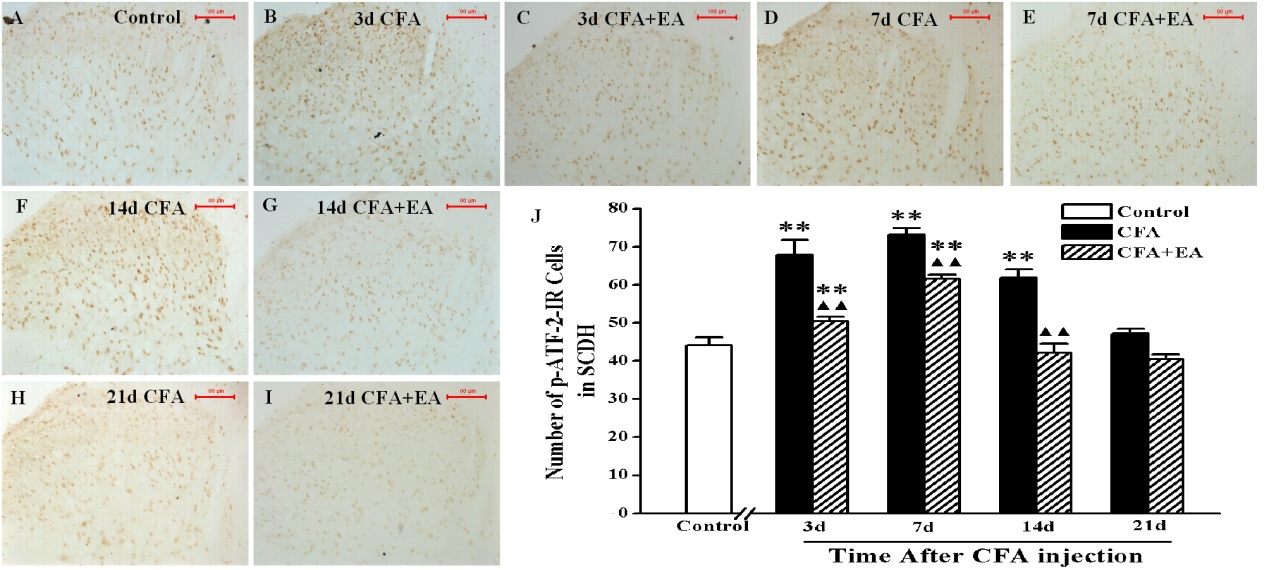


Supplementary figure 2


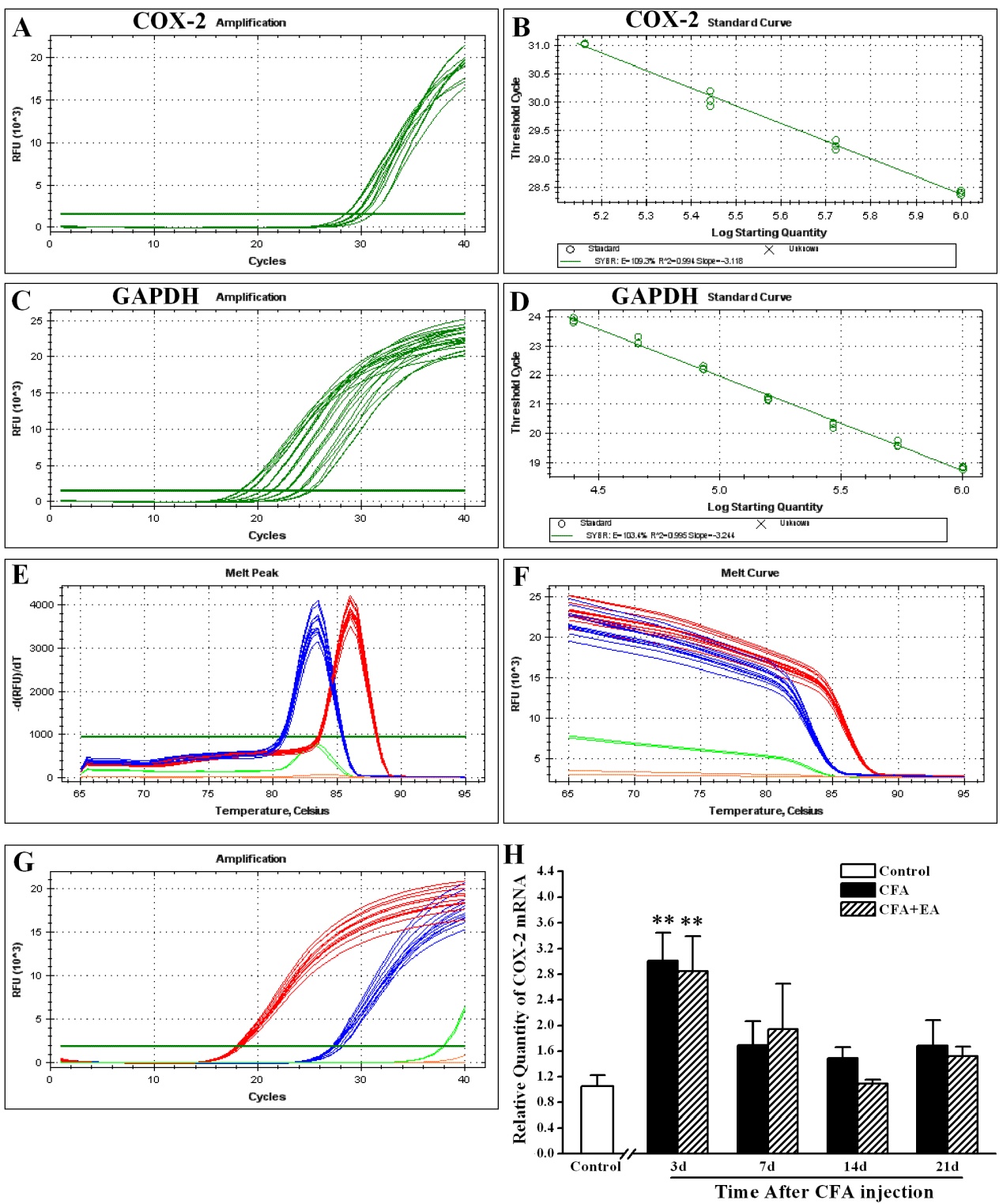


Supplementary figure 3


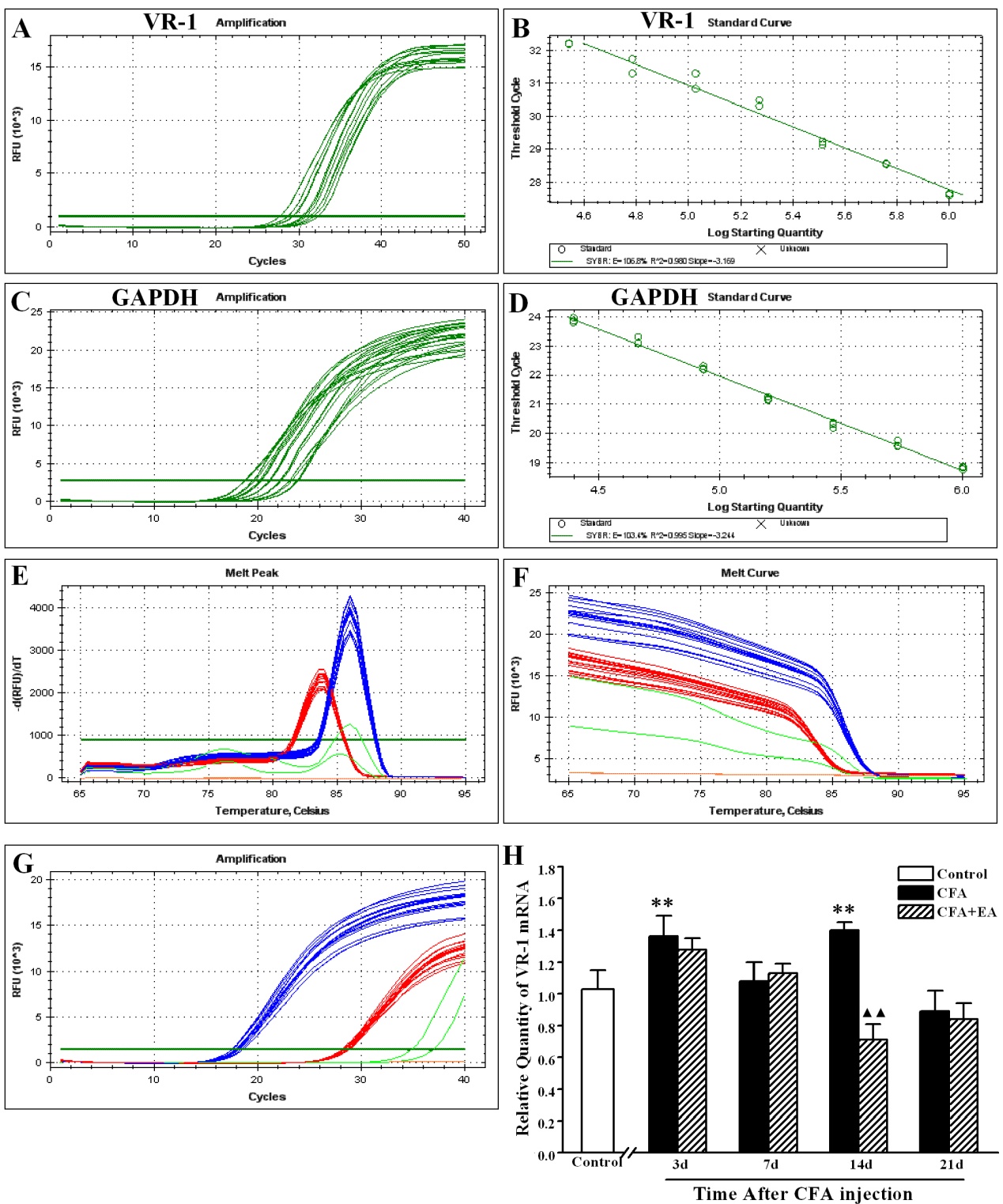


Supplementary figure 4


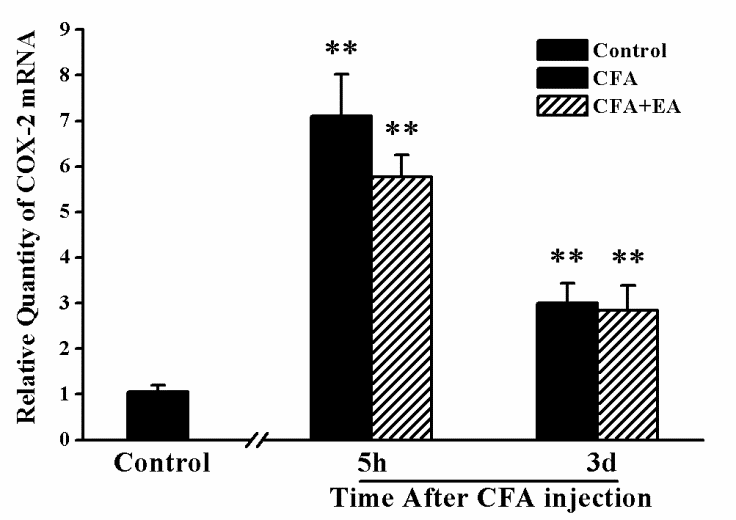


Supplementary figure 5

**Legend to supplementary figures**

**1. Supplementary figure 1- Effect of EA on p-p38MAPK expression in the ipsilateral spinal dorsal horn at different time points.**

The expression of p-p38 MAPK protein in spinal cord dorsal horn of the rats in control group and in CRA and CFA+EA group at 3 d, 7 d, 14 d and 21 d after CFA injection. A-I. Immunohistochemistry shows p-p38 MPAK-IR cells in the L4-L6 ipsilateral spinal cord dorsal horn in control rats (A), CFA rats at 3 d (B), 7 d (D), 14 d (F), 21 d (H), and CFA+EA rats at 3 d (C), 7 d (E), 14 d (G), 21 d (I). J. Quantification of p-p38 MAPK-IR cells in L4-L6 ipsilateral spinal cord dorsal horn. Results are shown as mean±SEM; n=3-6. ***P*<0.01 versus Control group at the corresponding time point. ^▲▲^*P*<0.01 versus CFA group at the corresponding time point.

**2. Supplementary figure 2-** **Effect of EA on p-ATF-2 expression in the ipsilateral spinal dorsal horn at different time points.**

The expression of p-ATF-2 protein in spinal cord dorsal horn of the rats in control group and in CRA and CFA+EA group at 3 d, 7 d, 14 d and 21 d after CFA injection. A-I. Immunohistochemistry shows p-ATF-2-IR cells in the L4-L6 ipsilateral spinal cord dorsal horn in control rats (A), CFA rats at 3 d (B), 7 d (D), 14 d (F), 21 d (H), and CFA+EA rats at 3 d (C), 7d (E), 14 d (G), 21 d (I). J. Quantification of p-ATF-2-IR cells in L4-L6 ipsilateral spinal cord dorsal horn. Results are shown as mean±SEM; n=3-6. ***P*<0.01 versus Control group at the corresponding time point. ^▲▲^*P*<0.01 versus CFA group at the corresponding time point.

**3. Supplementary figure 3- Effect of EA on COX-2 mRNA expression in the ipsilateral spinal dorsal horn at different time points.**

A-D) Standard curve of COX-2 and GAPDH mRNA in the ipsilateral spinal cord dorsal horn. Real-time PCR amplification of COX-2 mRNA (A) and GAPDH mRNA (C), standard curve of COX-2 mRNA (B) and GAPDH mRNA (D), the PCR efficiency (E) of COX-2 mRNA is 109.3%, slope is -3.118 and correlationcoefficient (R^2^) is 0.994, GAPDH mRNA’s E is 103.4%, slope is -3.244 and R^2^ is 0.995. E-G) amplification and melt curve of COX-2 and GAPDH mRNA in the ipsilateral spinal cord dorsal horn. Red represent GAPDH mRNA, blue represent COX-2 mRNA, green represent GAPDH mRNA non-sample control, yellow represent COX-2 mRNA non-sample control. H) Relative quantity of COX-2 mRNA transcription in the ipsilateral spinal cord dorsal horn at 3 d, 7 d, 14 d and 21 d after CFA injection in control, CFA, CFA+EA groups. EA down-regulate COX-2 mRNA expression in the ipsilateral SCDH. These data are displayed as mean±SEM, n=5-7. **P*<0.05, ***P*<0.01 versus Control group at the corresponding time point, ^▲^*P*<0.05 versus CFA group at the corresponding time point.

**4. Supplementary figure 4- Effect of EA on VR-1 mRNA expression in the ipsilateral spinal dorsal horn at different time points.**

A-D) Standard curve of VR-1 and GAPDH mRNA in the ipsilateral SCDH. Real-time PCR amplification of VR-1 mRNA (A) and GAPDH mRNA (C), standard curve of VR-1 mRNA (B) and GAPDH mRNA (D), the PCR efficiency (E) of VR-1 mRNA is 106.8%, slope is -3.169 and correlationcoefficient (R^2^) is 0.980, GAPDH mRNA’s E is 103.4%, slope is -3.244 and R^2^ is 0.995. E-G) amplification and melt curve of VR-1 and GAPDH mRNA in the ipsilateral spinal cord dorsal horn. Red represent GAPDH mRNA, blue represent VR-1 mRNA, green represent GAPDH mRNA non-sample control, yellow represent VR-1 mRNA non-sample control. H) Relative quantity of VR-1 mRNA transcription in the ipsilateral spinal cord dorsal horn at 3 d, 7 d, 14 d and 21 d after CFA injection in control, CFA, CFA+EA groups. EA down-regulate VR-1 mRNA expression in the ipsilateral SCDH. These data are displayed as mean±SEM, n=5-7. **P*<0.05, ***P*<0.01 versus Control group at the corresponding time point Control, ^▲^*P*<0.05 versus CFA group at the corresponding time point.

**5. Supplementary figure 5- Effect of EA on COX-2 mRNA expression in the ipsilateral spinal dorsal horn at 5h and 3d after CFA injection.**

The expression of COX-2 mRNA (relative to GAPDH) in L4-6 ipsilateral spinal dorsal horn measured by real-time PCR. ***P*＜0.01 versus control group. Results are mean±SEM; n=5-7.

**Supplemental results**

**1. Effect of EA on phosphor-p38 MAPK**

As shown in supplementary Fig. 1, peripheral inflammation induced by CFA injection resulted in the activation of p38 MAPK in the superficial dorsal horn on the ipsilateral side of the L4-L6 spinal cord. The number of phospho-p38 MAPK (p-p38 MAPK)-IR cells were increased at 3 d and with a slow decline for 14 d (supplementary Fig. 1I). However, the number of p-p38 MAPK-IR cells was recovered at 21 d after CFA injection. Then we tested the effect of EA on p38 MAPK activation at 3 d, 7 d, 14 d and 21 d after CFA injection. the increased numbers of p-p38 MAPK IR cells in the superficial spinal cord dorsal horn (SCDH) was suppressed by the EA treatment at 3 d, 7 d and 14 d after CFA injection (*P*<0.01).

**2. Effect of EA on phosphor-ATF-2**

We further investigated expressions of p-ATF-2, a downstream material of p3 MAPK, in superficial dorsal horn. As shown in supplementary Fig. 2, the peripheral inflammation induced by CFA injection resulted in the high expression of ATF-2-IR cells in the ipsilateral dorsal horn on the L4-L6 spinal cord during the 3 d to 14 d after CFA injection. We also tested the effect of EA on ATF-2 activation at 3 d, 7 d, 14 d and 21 d after CFA injection. As the same as p38 MAPK, the increased numbers of p-ATF-2-IR cells in the superficial SCDH were suppressed by the EA treatment at 3 d, 7 d and 14 d after CFA injection (*P*<0.01).

**3. Effect of EA on COX-2 mRNA**

To investigate whether peripheral inflammation or EA could regulate COX-2 expression in SCDH, we used real-time PCR to test the COX-2 mRNA at 3 d, 7 d, 14 d and 21 d after CFA treatment. As shown in supplementary Fig. 3, compared with that of control group, COX-2 mRNA expression in SCDH only increased at 3 d after CFA injection (*P*<0.01). However, EA treatment didn't show any inhibiting effect on CFA-induced over-expression of COX-2 mRNA at that time point (*P*>0.05).

In another study of our lab, we tested the effect of peripheral inflammation and EA on regulation COX-2 mRNA expression in SCDH at 5 h and 3 d after CFA injection. COX-2 mRNA expression in SCDH increased both at 5 h and 3 d after CFA administration, compared with that of control group. However, EA treatment did not show any inhibition effect on COX-2 mRNA both at 5h and 3d after CFA injection (supplementary Fig. 5).

**4. Effect of EA on VR-1 mRNA**

We also investigated the effect of peripheral inflammation and EA on induction VR-1 mRNA in SCDH by using real-time PCR technology. The results show in supplementary Fig. 4. Higher expression of VR-1 mRNA in SCDH was induced by peripheral inflammation at both 3 d and 14 d after CFA injection, compared with that of control group. EA treatment significantly inhibited CFA-induced over-expression of VR-1 mRNA at 14 d after CFA injection. However, EA only show a trend of inhibition VR-1 mRNA expression at 3 d after CFA injection.
